# Psychosocial oral health‐related quality of life impact: A systematic review

**DOI:** 10.1111/joor.13064

**Published:** 2020-08-14

**Authors:** Naichuan Su, Arjen van Wijk, Corine M. Visscher

**Affiliations:** ^1^ Department of Social Dentistry Academic Center for Dentistry Amsterdam (ACTA) University of Amsterdam and VU University Amsterdam Amsterdam The Netherlands; ^2^ Department of Orofacial Pain and Disfunction Academic Center for Dentistry Amsterdam (ACTA) University of Amsterdam and VU University Amsterdam Amsterdam The Netherlands

**Keywords:** dental anxiety, oral cancer, Oral Health‐Related Quality of Life, periodontitis, psychosocial impact

## Abstract

**Background:**

Psychosocial wellbeing is an important determinant for patients' oral health‐related quality of life (OHRQoL). Psychosocial impact (PI), together with the dimensions Oral Function, Orofacial Pain and Orofacial Appearance, has been proposed to cover the different areas of OHRQoL.

**Objective:**

The objective of the study was to collect further scientific support for the new four‐dimensional structure of OHRQoL. This study is one out of a series of four and focuses on the PI in patients with dental anxiety, oral cancer and periodontitis (PROSPERO registration number: CRD42017064033).

**Methods:**

Five databases (Pubmed (Medline), EMBASE, Cochrane, CINAHL and PsycINFO) were electronically searched on 8 June 2017 and updated on 14 January 2019, to identify the studies that measure OHRQoL using the Oral Health Impact Profile (OHIP) for oral health conditions. In this review, studies were included if the mean/median domain scores from OHIP‐14 or OHIP‐49 were available for patients with dental anxiety, oral cancer or periodontitis. The score of the handicap domain from the OHIP was used to assess patients` PI. The handicap domain includes 6 items for OHIP‐49 with a domain score ranging from 0 to 24 and 2 items for OHIP‐14 with a domain score ranging from 0 to 8. For comparison between the 2 versions of the OHIP, the domain score of OHIP‐49 was conversed into a 0 to 8 metric. The domain scores of the included studies were then pooled, separately for each of the included dental disorders.

**Results:**

A total of 2104 records were identified based on the search strategy. After screening of titles and abstracts, 1607 articles were reviewed in full text. Twenty‐three articles met the inclusion criteria for this review and were included in the study. The 23 articles contained 3884 patients, grouped in 30 patient populations and 42 patient samples. The pooled mean scores of PI for dental anxiety, oral cancer and periodontitis were 3.2, 1.9 and 0.8, respectively, on the 0 to 8 metric.

**Conclusion:**

This review provides standardised information about the OHRQoL impact for three dental disorders as a model for the PI dimension. Dental anxiety tends to show the strongest effect on the PI dimension, while periodontitis tends to show the weakest effect on the PI dimension. Future studies need to confirm whether the reported differences in PI scores between the three dental disorders are statistically significant.

## 1 INTRODUCTION

In daily practice, a dentist needs to objectify a patient's complaints both on a physical level and on a psychosomatic level. Based on this information, a patient‐tailored treatment plan can be developed, taking into account patient characteristics that may interfere with healing and/or treatment adherence.^1^ Clinicians in most (if not all) medical fields accept the biopsychosocial model as the most heuristic approach to understand and manage (chronic) pain and dysfunction. In this model, pain and disability interact with psychological and social factors, and these factors together determine the impact of a clinical condition on the individual patient.^2^ In the field of dentistry, this dual‐axis approach is incorporated in the diagnostic classification for temporomandibular disorder patients^3^ and could be applied to other pain conditions, including the various dental pain patients.^1^ Options to measure psychological and social factors include questionnaires on specific constructs, like depression,^4^ anxiety^5^ or social support.^6^

During the treatment process, the outcomes of treatment are continuously monitored and compared with the expectations for that treatment. Patient‐perceived impact of treatment nowadays is considered a highly important tool to evaluate treatment success. To measure the patient‐perceived impact consistently across different oral health conditions, the concept of Oral Health‐Related Quality of Life (OHRQoL) is widely acknowledged. The most commonly used questionnaire to measure OHRQoL is the Oral Health Impact Profile (OHIP).^7^ The OHIP was originally developed and evaluated by Slade and Spencer in 1994.^7^ Up to date, the OHIP has been translated into multiple languages and is further developed into several versions with a smaller number of items as compared to the original 49‐item of the OHIP, such as the 14‐item version.^7^, ^8^ In both OHIP‐14 and OHIP‐49, seven domains of OHRQoL are incorporated, covering functional limitation, physical pain, psychological discomfort, physical disability, psychological disability, social disability and handicap. The OHIP can be used to capture changes in health, with a proposed 7‐day recall period.^9^ In clinical practice and research, both OHIP‐14 and OHIP‐49 are widely used for the assessment of OHRQoL in different target populations. However, OHIP‐14 is preferred to OHIP‐49 by researchers and clinicians because it is more practical due to less number of items, while it still has acceptable reliability, validity and precision.^8^

Recent empirical data have shown that an approach using only four dimensions (ie *Orofacial Pain, Orofacial Appearance, Oral Function and Psychosocial Impact*) can serve as a more simple and clinically appealing set of OHRQoL dimensions and still provide a psychometrical accurate OHRQoL measurement.^10^ In a recent systematic review, it was found that the four OHRQoL dimensions were the attributes that underlie all generic dental patient‐reported outcome measures.^11^

In this special issue, the utility of the four‐dimensional structure of OHRQoL is evaluated.^10^ In a series of four systematic reviews, one for each dimension, further evidence for the concept of these new dimensions is sought. Therefore, the aim of this systematic review is to collect further scientific support for the new four‐dimensional structure by specifically describing the psychosocial impact (PI) on OHRQoL in dental patients. For this purpose, dental patient populations with presumed elevated levels of PI, as well as dental patient populations with more equally distributed impacts on the four dimensions, were included in the review (ie patients with dental anxiety, oral cancer and periodontitis).

## 2 MATERIALS AND METHODS

The protocol that was used in this systematic review was registered in PROSPERO (CRD42017064033) and was carried out in accordance with the Preferred Reporting Items for Systematic Reviews and Meta‐Analyses (PRISMA) Statement.

### 2.1 Subjects and outcome variable of the study

The target populations selected for this review were patients with dental anxiety, oral cancer or periodontitis. These patient groups were selected, because patients with dental anxiety were assumed to be mostly affected on the psychosocial aspect of OHRQoL, relative to the other dimensions, while patients with oral cancer and patients with periodontitis were assumed to be more equally affected on the four dimensions.^12^

To assess the PI of the patient group, both publications using the OHIP‐49^7^ and the OHIP‐14^8^ questionnaire were used. As proposed in the four‐dimensional structure for the description of OHRQoL, the handicap domain from the OHIP was used to assess patients’ PI in the present study.^12^ In other words, the two items from the handicap domain of OHIP‐14 or the six items from the same domain of OHIP‐49 were used to assess the PI dimension.

### 2.2 Literature search

A review of the literature was conducted by a trained librarian (NTM, see Acknowledgements) who utilised natural language to identify all articles that measure OHRQoL, for any oral health condition, using the OHIP. The keywords used to retrieve the articles were ‘Oral Health Impact Profile’ or ‘OHIP’. The searches were performed in PUBMED, EMBASE, Cochrane, CINAHL and PsyINFO on 8 June 2017 and were updated in 14 January 2019. EndNote was used to remove any duplicate publications. Grey literature was not included, and authors were not contacted for additional information. For more detail on the literature search, see the methods chapter.^12^

### 2.3 Selection criteria and study screening

Two reviewers (SS, NTM, see Acknowledgements) independently assessed the titles and abstracts of all identified studies from the electronic searches. SS and MTJ (see Acknowledgements) then determined whether an article fulfilled the criteria to be assigned to the review on the PI dimension. The inclusion and exclusion criteria for the general screening are described in the methods chapter.^12^ Then, two authors (AvW and NS) of the PI dimension screened the full‐text articles based on the specific inclusion and exclusion criteria of the PI dimension presented below. In case of disagreement, a third author (CV) was added for a majority vote.

The inclusion criteria for an article to be included in the review on the PI dimension were as follows:

1. The OHIP‐49 or the OHIP‐14 was used for the assessment of OHRQoL;
2. The study sample included patients with dental anxiety, oral cancer or periodontitis;
3. Scores for each domain of the OHIP were reported or could be calculated;
4. Scores for each domain summarised using additive scores (OHIP‐ADD) method.^13^ The OHIP‐ADD method is defined as the sum score of the OHIP calculated by summing the score of each item of the OHIP. The score of each item ranges from 0 to 4 and thereby the sum scores of the OHIP‐14 and the OHIP‐49 range from 0 to 56 and 0 to 196, respectively. The sum scores of the handicap domain, which was used for the PI dimension, range from 0 to 8 for the OHIP‐14 and from 0 to 24 for the OHIP‐49. Studies in which the OHIP‐ADD method was used, with the individual item scores ranging from 1 to 5, were also included and converted to match the OHIP scores as based on 0 to 4 scores.

The exclusion criteria for papers in this systematic review on the PI dimension were as follows:

1. Full‐text not available;
2. Non–English‐language articles;

### 2.4 Data extraction

For all included studies, the following data were extracted using a standardised form: (a) first author`s name; (b) year of publication; (c) name of the journal; (d) population of the study subjects; (e) current disease conditions of the study subjects; (f) country, (g) version of the OHIP; (h) study design (follow‐up study/cross‐sectional study); (i) proportion of male/female; (j) mean and median age with the standard deviation (SD), interquartile range (IQR), age range and 95% confidence interval (CI); (j) mean and median of the OHIP domain scores and sum scores with measures of dispersion including SD, IQR, range and 95% CI.

For included articles which had more than one patient sample, the characteristics of patients in each sample were extracted separately.

### 2.5 Quality assessment

The methodological quality of included studies was assessed based on a 10‐item appraisal tool for prevalence studies.^14^ The risk of bias was assessed separately for each patient sample of the included studies.

Six of the 10 items were deemed useful for the risk of bias assessment of the included studies, that is representativeness of the samples, recruitment, characterisation of the subjects and the settings, coverage of the samples in the data analysis, standard criteria for measurement of the conditions and reliability of measurement of the conditions. The answer to each question is ‘Yes’, ‘No’ or ‘Unclear’. A ‘risk of bias’ judgment (‘low’, ‘unclear’ or ‘high’) was made for each signalling question. If the answer to a signalling question was judged as ‘Yes’, it was judged as ‘low risk’ of bias for this question. If the answer to a signalling question was judged as ‘No’, it was judged as ‘high risk’ of bias for this question. Otherwise, the question was judged as ‘unclear risk’. For more detail on quality assessment, see the methods chapter.^12^

The quality assessment was performed independently by three authors (NS, CV and AvW), and consensus was reached through discussion.

### 2.6 Data synthesis and analysis

To enhance comparison of data between papers that used different versions of the OHIP, mean domain scores and 95% CI values of studies using the OHIP‐49 were converted to match mean and 95% CI of the OHIP‐14. After conversion, the mean score on each domain ranges from 0 to 8. If the mean values were not provided in the original paper, median values were used for the data analysis. If the 95% CI values were not given, they were calculated from SD values, stand error (SE) values, IQR values, or first and third quartile range values. For details, see the methods chapter.^12^

For each dental disorder included in the PI dimension (dental anxiety, oral cancer and periodontitis), the pooled mean score was calculated by pooling the mean scores of the corresponding samples of patients. The pooled mean scores of each disorder were calculated weighted by the sample size of the patient populations in the included studies.

## 3 RESULTS

### 3.1 Results of search and selection

**FIGURE 1:**
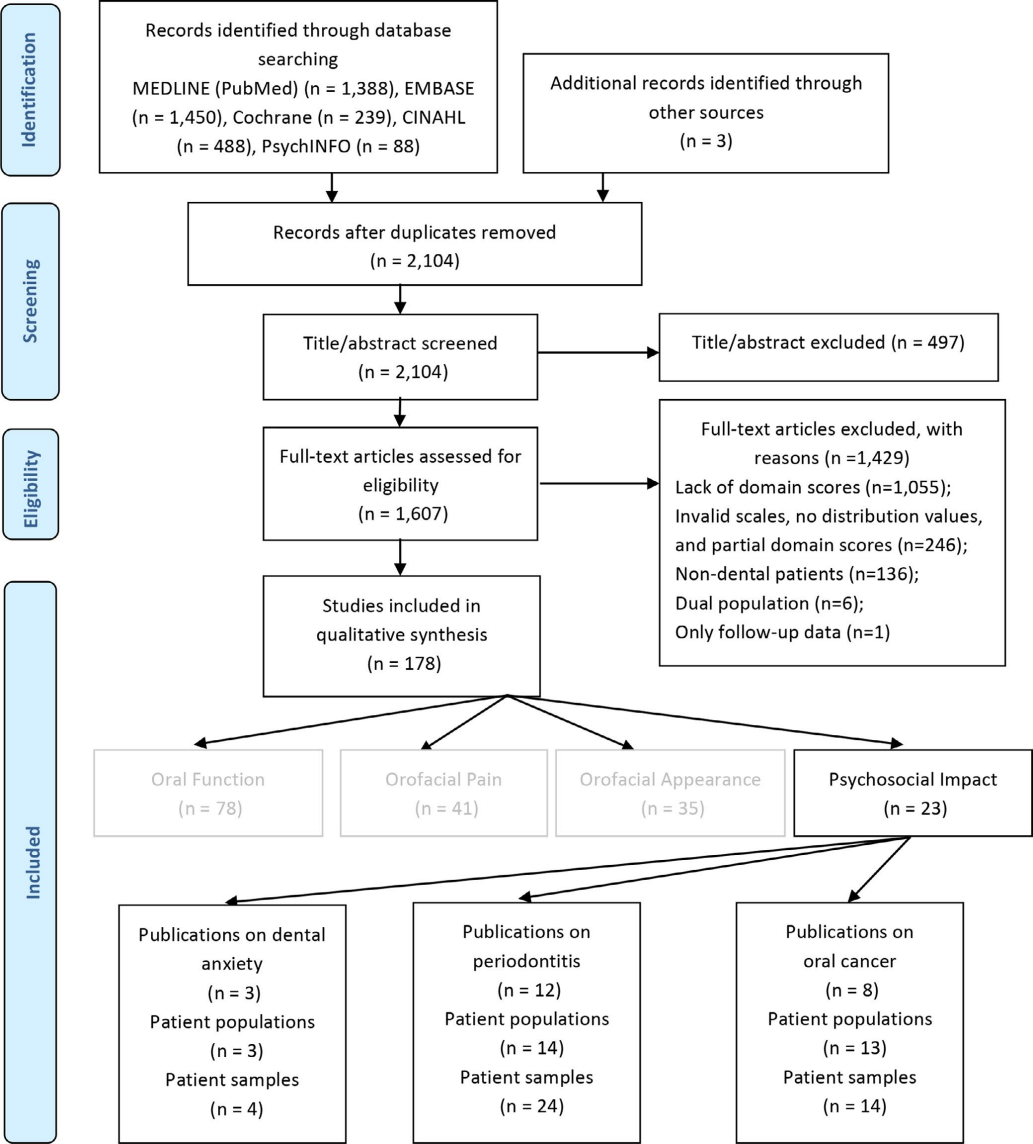
PRISMA flowchart of the inclusion process of publications in the review on the Psychosocial Impact dimension

The initial search identified a total of 2104 studies.^12^ After screening of the titles, abstracts and full texts, 23 studies were included in the present review (Figure 1).^15^, ^16^, ^17^, ^18^, ^19^, ^20^, ^21^, ^22^, ^23^, ^24^, ^25^, ^26^, ^27^, ^28^, ^29^, ^30^, ^31^, ^32^, ^33^, ^34^, ^35^, ^36^, ^37^

### 3.2 Characteristics of included studies

The 23 included studies contained a total of 30 patient populations and 42 patient samples.^15^, ^16^, ^17^, ^18^, ^19^, ^20^, ^21^, ^22^, ^23^, ^24^, ^25^, ^26^, ^27^, ^28^, ^29^, ^30^, ^31^, ^32^, ^33^, ^34^, ^35^, ^36^, ^37^ Among the studies, 12 studies (24 samples) involved periodontal patients,^26^, ^27^, ^28^, ^29^, ^30^, ^31^, ^32^, ^33^, ^34^, ^35^, ^36^, ^37^ 8 studies (14 samples) involved oral cancer patients^18^, ^19^, ^20^, ^21^, ^22^, ^23^, ^24^, ^25^ and 3 studies (4 samples) involved anxiety patients.^15^, ^16^, ^17^ The 23 studies were conducted in 10 countries. Most studies were performed in China (N = 5), India (N = 3), Brazil (N = 3) and Israel (N = 3). Four of the studies used the OHIP‐49 for patients’ OHRQoL while the others used the OHIP‐14. The sample characteristics are presented in more detail in Table 1.

**Table 1:** OHIP scores for included patient samples in the Psychosocial Impact dimension

Year	Population	Population, N (% women)	Mean ages (SD), Range	Instrument Original mean score (SD)	Standardised mean score (95%CI)^b^
*Dental anxiety*					
Almoznino 2015^15^	I: Dental anxiety II: Gag reflex	68 (36.8%) 54 (35.2%)		OHIP‐14 4.46 (2.18) OHIP‐14 4.47 (2.25)	4.46 (3.93‐4.99) 4.47 (3.86‐5.08)
Vermaire 2008^16^	I: Dental anxiety‐routine care	33 (49%)	34.1 (9.2) 18‐55	OHIP‐14 2.07 (1.15)	2.07 (1.66‐2.48)
Vermaire 2016^17^	I: Dental anxiety	76 (42.1%)	42.6 (11.9) 25‐71	OHIP‐14 1.6 (0‐4)^a^	1.6 (0.37‐2.83)
*Oral cancer*					
Barrios 2015a^18^	I: Oral cancer survivors prior to prosthetic	142 (35.9%)	65.2 (12.9)	OHIP‐14 2.1 (2.1)	2.1 (1.75‐2.45)
Dholam 2016^19^	I: Prior to rehabilitation before obturator II: Prior to rehabilitation before partial denture III: Prior to rehabilitation before complete denture	45 (42.2%) 20 (25%) 10 (10%)	43 52 62	OHIP‐14 1.21 (1.27) OHIP‐14 1.10 (1.26) OHIP‐14 0.50 (0.75)	1.21 (0.83‐1.59) 1.10 (0.51‐1.69) 0.50 (0.00‐1.03)
Dholam 2017^20^	I: Prior to prosthetic rehabilitation (no treatment)	60 (31.7%)	53 14‐73	OHIP‐14 0.73 (1.09)	0.73 (0.45‐1.01)
Indrapriyadhar shini 2017^21^	I: Treated by surgery and radiotherapy II: Treated by surgery alone III: Treated by surgery, chemotherapy, and radiotherapy	30 (53.33%) 30 (76.67%) 30 (20%)		OHIP‐14 4.64 (2.82) OHIP‐14 4.07 (2.67) OHIP‐14 5.37 (2.42)	4.64 (3.59 5.69) 4.07 (3.07 5.07) 5.37 (4.47 6.27)
Li 2017^22^	I: Head and neck cancer, survivors	77 (32.5%)	67.7 (10.66) 26‐86	OHIP‐14 1.6 (1.6)	1.6 (1.24 1.96)
McMillan 2004^23^	I: NPC, new diagnosis II: NPC, survivors	40 (17%) 38 (29%)	46.7 (10.2) 50.1 (10.1)	OHIP‐49 1.1 (0.34) OHIP‐49 3.8 (0.81)	0.44 (0.40 0.48) 1.52 (1.41‐1.63)
Pow 2012^24^	I: NPC, radiation	58 (40.6%)	46.9 (9.5) 28‐70	OHIP‐49 0.9 (1.6)	0.36 (0.19 0.53)
Stuani 2018^25^	I: Head and neck cancer, prior to treatment II: Post‐radiotherapy and/or chemotherapy phase	20 (25%) 20 (15%)	53.75 (16.91) 58.3 (10.63)	OHIP‐14 1.27 (1.23) OHIP‐14 1.42 (1.21)	1.27 (0.69 1.85) 1.42 (0.85 1.99)
*Periodontitis*					
Desai 2014^26^	I: Periodontitis: manual self‐complete II: Periodontitis: tel. interview			OHIP‐49 0 (0‐3)^a^ OHIP‐49 1 (0‐4)^a^	0 (0.00 0.17) 0.4 (0.18 0.62)
Durham 2013^27^	I: Chronic periodontitis	89	47 (9)	OHIP‐49 2.76 (3.66)	1.10 (0.80‐1.41)
Goh 2018^28^	I: Aggressive periodontitis, treated	89 (58.4%)	25.2 (3.2) 18‐30	OHIP‐14 1.1 (1.3)	1.1 (0.83 1.37)
Al Habashneh 2012^29^	I: Periodontitis, mild II: Gingivitis, chronic III: Periodontitis, moderate IV: Periodontitis, severe	79 167 93 61		OHIP‐14 1.03 (1.24) OHIP‐14 1.14 (1.44) OHIP‐14 1.78 (1.66) OHIP‐14 2.11 (1.74)	1.03 (0.75 1.31) 1.14 (0.92 1.36) 1.78 (1.44 2.12) 2.11 (1.66 2.56)
Ilanos 2018^30^	I: Generalised aggressive periodontitis II: Generalised chronic periodontitis III: Localised aggressive periodontitis	33 (66.6%) 10 (40%) 9 (77.7%)	30.7 (5) 50.1 (6.8) 25.5 (7.4)	OHIP‐14 0.8 (1.2) OHIP‐14 0.7 (1.0) OHIP‐14 0.2 (0.7)	0.8 (0.37 1.23) 0.7 (0.00 1.42) 0.2 (0.00 0.74)
Jansson 2014^31^	I: Bone loss > 1/3 root length, < 30% teeth II: Bone loss < 1/3 root length III: Bone loss > 1/3 root length,> 30% teeth	90 (56%) 304 (52%) 49 (41%)	59.9 (11.4) 42.5 (15.4) 64.4 (11.8)	OHIP‐14 0.41 (1) OHIP‐14 0.37 (0.87) OHIP‐14 1.23 (1.89)	0.41 (0.20‐0.62) 0.37 (0.27‐0.47) 1.23 (0.69‐1.77)
Kato 2018^32^	I: Periodontitis, 70‐year‐old women II: Periodontitis, 70‐year‐old men III: Periodontitis, 78‐year‐old women IV: Periodontitis, 82‐year‐old women	303 (100%) 235 (0%) 148 (100%) 118 (100%)	70 70 78 ≥82	OHIP‐14 0.3 (1.0) OHIP‐14 0.3 (0.9) OHIP‐14 0.4 (1.0) OHIP‐14 0.3 (0.8)	0.3 (0.15 0.45) 0.3 (0.18 0.42) 0.4 (0.24 0.56) 0.3 (0.19 0.41)
Levin 2018a^33^	I: Chronic periodontitis	99 (18.2%)	38.8 (7.8)	OHIP‐14 0.69 (0.85)	0.69 (0.55 0.83)
Levin 2018b^34^	I: Aggressive periodontitis	60	24.7 (7.7)	OHIP‐14 0.83 (1.03)	0.83 (0.56 1.10)
Lu 2016^35^	I: Halitosis group	102 (55.9%)	37.7 (14.2)	OHIP‐14 2.1 (1.7)	2.1 (1.77 2.43)
Mendez 2017^36^	I: Gingivitis, moderate/severe periodontitis	55 (65.5%)	51.4 (9.4)	OHIP‐14 1.4 (1.7)	1.4 (0.94 1.86)
Ng 2006^37^	II: Periodontitis, low/control I: Periodontitis, severe	584 143		OHIP‐14 0.28 (0.6) OHIP‐14 0.33 (0.52)	0.28 (0.23 0.33) 0.33 (0.24 0.42)

Abbreviations: 95% CI: 95% confidence interval; NPC, Nasopharyngeal carcinoma; OHIP, Oral Health Impact Profile; SD, Standard deviation.

^a^ Median (interquartile range);

^b^ Mean scores and SD from OHIP‐49 were converted into OHIP‐14 mean and 95%CI values.

### 3.3 Quality assessment

**FIGURE 2:**
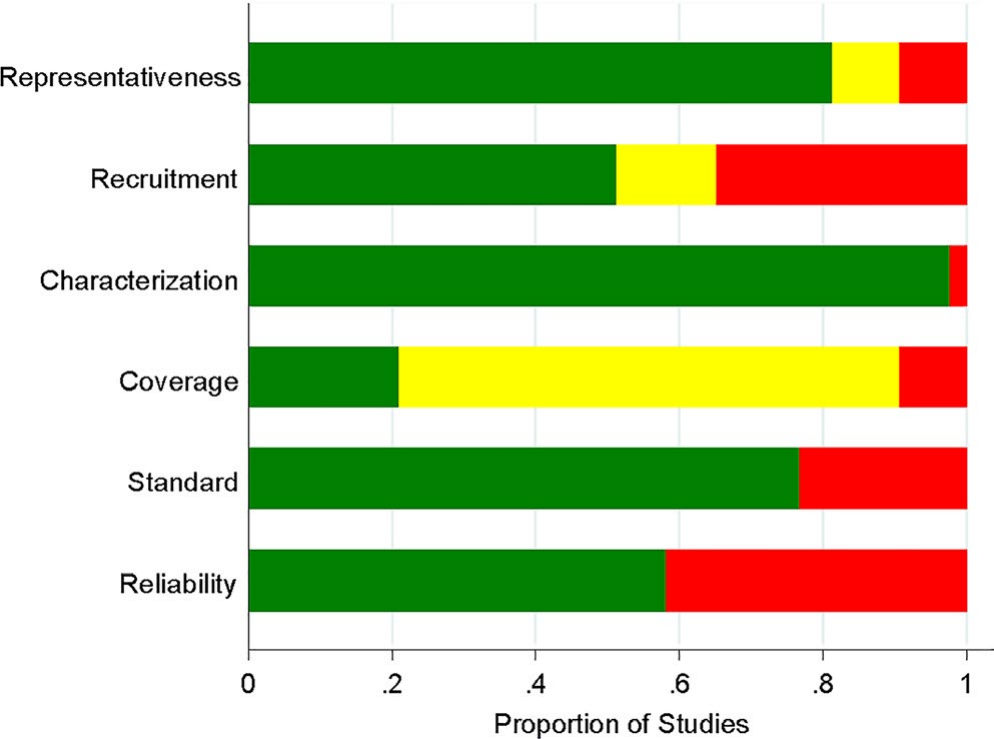
Risk of bias assessment of patient samples of included studies based on review authors` judgments about each risk of bias item presented as percentages across all included studies. Green indicates low risk of bias. Yellow indicates unclear risk of bias. Red indicates high risk of bias

Most patient samples from the studies showed high methodological quality in most categories of the tool (see Figure 2). For representativeness, 4 samples from 2 studies were regarded as having a ‘high risk’ of bias,^16^, ^21^ because the patient populations did not match the target patients of these studies. Four samples from another 2 studies were regarded as having ‘unclear risk’ of bias,^19^, ^20^ because no sufficient information on the source of included patients was provided. For recruitment, 15 samples from 8 studies were regarded as ‘high risk’ of bias,^15^, ^16^, ^21^, ^22^, ^26^, ^28^, ^30^, ^37^ because the patients’ recruitment phase was short (< 6 months), the sample size was small (<100 patients) or convenience sampling was used to recruit patients. Six samples from another 4 studies were regarded as ‘unclear risk’ of bias,^18^, ^20^, ^23^, ^25^ because of insufficient information on the type of sampling, period of time of recruitment or sample size. For characterisation, 1 sample from 1 study was regarded as ‘high risk’ of bias,^20^ because the description of the characteristics of clinical settings was insufficient while no samples were regarded as ‘unclear risk’ of bias. For coverage, 4 samples from 2 studies were regarded as ‘high risk’ of bias,^28^, ^31^ because patients’ response rate was too low, while 31 samples from another 14 studies were regarded as ‘unclear risk’ of bias,^15^, ^17^, ^19^, ^20^, ^21^, ^23^, ^25^, ^26^, ^27^, ^29^, ^30^, ^32^, ^35^, ^36^ because patients’ response rate was not reported and could not be calculated. For standard, 10 samples from 5 studies were regarded as ‘high risk’ of bias,^16^, ^19^, ^20^, ^21^, ^25^ because the language‐version of the OHIP used in the studies was not reported or the language‐version of the OHIP used was not validated in previous publications. No samples were regarded as having ‘unclear risk’ of bias. For reliability, 18 samples from 11 studies were regarded as ‘high risk’ of bias,^15^, ^16^, ^19^, ^20^, ^21^, ^22^, ^24^, ^25^, ^26^, ^36^ because the used measurement for OHRQoL in these studies was not standardised and the objective of the OHIP was not self‐reported by the patients (though with the assistance of others). No samples from the studies were regarded as ‘unclear risk’ of bias in this category (Figure 2).

### 3.4 Results of data synthesis and analysis

**FIGURE 3:**
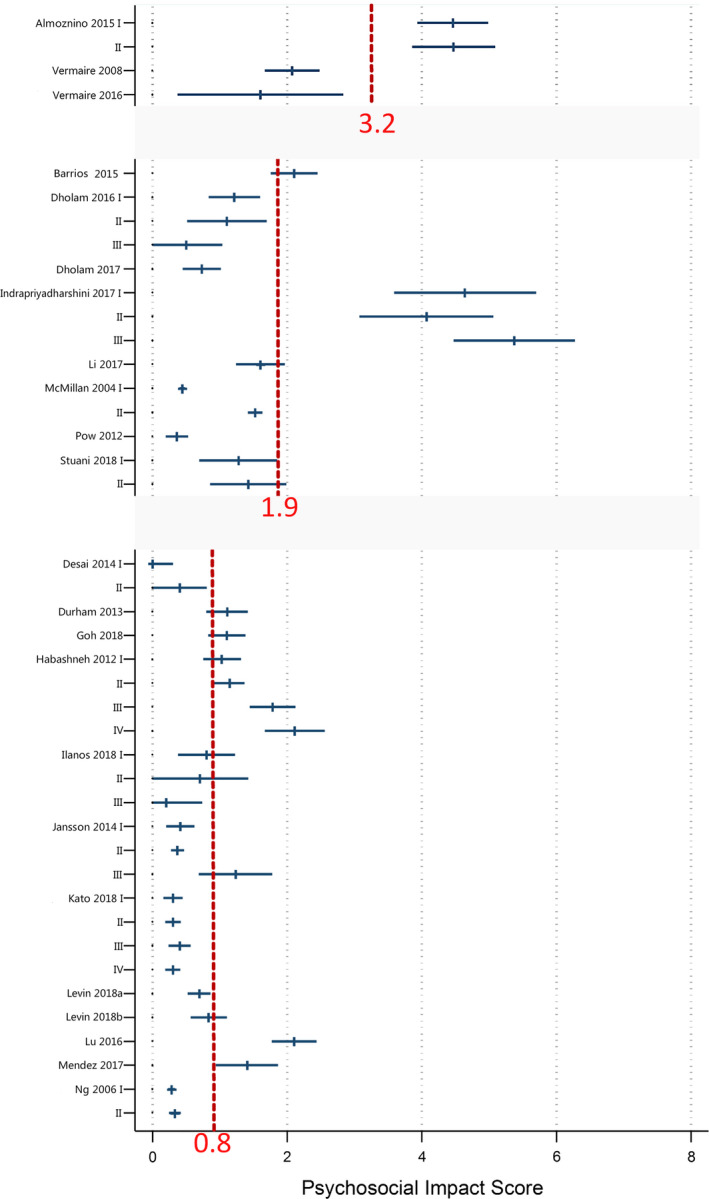
Forest plots for the mean scores of included patient samples in the Psychosocial Impact dimension based on different disease disorders. The red dotted lines represent the pooled mean scores (weighted by sample size) of each disease disorder

The mean scores on the PI dimension of the individual samples of patients are presented in Figure 3. It shows that patients with dental anxiety, patients with a gag reflex and patients with oral cancer treated with surgery alone, surgery and radiotherapy, or a combination of surgery, radiotherapy and chemotherapy, had significantly higher mean scores (as based on their 95%CI values) on the PI dimension as compared to the other patients samples. The mean scores for those five patient samples ranged between 4 and 5. The mean scores in the remaining samples of patients ranged between 0 and 3.

The pooled mean score for dental anxiety patient samples (ie 3.2) was the highest, while the pooled mean score for periodontitis patient samples (ie 0.8) was the lowest. The pooled mean score for oral cancer patient samples was 1.9. This suggests that patients with dental anxiety perceive the strongest effect on the PI dimension, while patients with periodontitis perceive the weakest effect on the PI dimension, among these three types of dental disorders.

## 4 DISCUSSION

The aim of the present systematic review was to collect further scientific support for the new four‐dimensional structure of OHRQoL by specifically describing the PI dimension in the three dental patient populations. The samples consisted of patients with dental anxiety, oral cancer or periodontitis. A total of 23 studies were included, which covered 42 samples of patients, including 24 samples of periodontal patients, 14 samples of cancer patients and 4 samples of dentally anxious patients. Regarding the quality of the included studies, the majority showed high methodological quality on 3 specific categories (representativeness, characterisation and standard). On 2 categories (recruitment and reliability) a modest quality was observed, while the lowest quality was scored on the category coverage, with a large number of the samples rated as ‘unclear’. All in all, this suggests that the included studies were of adequate methodological quality. The present review provides standardised information regarding three dental patient populations that can be used as reference for the PI dimension within the newly proposed OHRQoL four‐dimensional structure.

Results showed that the pooled mean scores on the PI domain were relatively low for the sample of periodontitis (0.8) and oral cancer patients (1.9). In comparison, the sample of dentally anxious patients scored significantly higher (3.2). In other words, patients with dental anxiety perceived a stronger impact on the PI dimension of their OHRQoL than those with periodontitis or oral cancer. One explanation for this surprising outcome (especially for the relatively low score of the patients with oral cancer) resembles something referred to as the ‘disability paradox’. This paradox refers to the observation that many people with serious and persistent disabilities report that they experience a good or excellent quality of life while to most external observers these individuals seem to live an undesirable daily existence.^38^ Something similar might hold true for the oral cancer patients. For instance, some of these patients require invasive surgery, which can result in severe aesthetic impairment and a plausible subsequent impact on their psychosocial wellbeing (social isolation, feelings of shame, depressed, being stared at, etc). On the other hand, oral cancer patients also face a potentially life‐threatening condition which can put things into a different perspective. Especially when facing or having faced death, people tend to reconsider what they value as important, which is often family, friends and loved ones. So, contrary to the ‘plausible impact’ just mentioned, oral cancer patients may actually experience an increase in the quality of their social relations since these are valued higher. Social interactions may also be intensified given the life‐threatening condition these patients suffer from. Moreover, having survived cancer can give new meaning to live. Any possible aesthetic impairment as mentioned above can therefore seem relatively less important than before.

Periodontal problems, in the broadest sense of the word, were expected to impact the psychosocial dimension in a different way. For instance, the PI could be elevated because of possible impaired aesthetics due to missing teeth, or bad breath, perhaps hindering patients in their interaction with others. The results, however, do not support a strong impact on the PI dimension for patients with periodontitis. In fact, the category of periodontal patients, which contained the highest number of patients and samples, shows a consistent result regarding a relatively low mean score on the psychosocial domain.

As illustrated in Figure 3, large heterogeneity was found across the studies included in each of the dental disorder groups. One of the reasons for the large heterogeneity may be that individuals` self‐perception of their psychosocial wellbeing is a relatively subjective and unstable experience, which could be impacted by cultural norms of a society.^39^ Culture influences the way individuals think about, express and define their worlds.^40^ Individuals` perception of quality of life therefore depends on the cultural context and values of the system in which they live, and is associated with their goals, expectations, standards and concerns.^41^ Therefore, patients from different cultures may score differently on the PI dimension for equally serious disorders. Another reason for the large heterogeneity is that the included patients may differ in the severity of the disorder. For example, in the paper by Al Habashneh et al,^29^ patients with severe periodontitis scored twice as high on the PI dimension as compared to the patients with mild periodontitis.

One limitation of the present review concerns the fact that not all available studies could be included. For example, one study used a unique scoring system in which the domains of OHIP‐14 were scored from 0 to 100.^42^ Another study^43^ did not use the OHIP‐ADD method to calculate the sum score of the OHIP‐14. Instead, the OHIP simple counting (OHIP‐SC) scoring method was used. The OHIP‐SC is defined as the sum score of the OHIP calculated by the number of the items of the OHIP with impacts reported at a threshold level (for example, ‘fairly often’ or ‘very often’).^44^ Therefore, these data were not suitable for synthesis with results from the articles included in the present study. For more detailed information regarding the measurement of OHRQoL and its related practical issues please see the chapter by Reissman.^45^

In addition, only studies published in English were eligible to be included, and the grey literature was not investigated. Also, even though multiple versions of the OHIP exist, only the OHIP‐14 and the OHIP‐49 were included, while other versions of the OHIP, for instance, OHIP‐EDENT which was tailored for patients with prosthetics or edentulous patients,^46^ were excluded. It is complex to argue which specific effect these limitations may have had on the outcomes of this review.

Another limitation is related to grouping of different types of patients into one dental patient category. Consider for instance the periodontal patients, a category that contains patients with many different types of problems such as halitosis, (chronic) gingivitis, (chronic/aggressive) periodontitis or (mild/moderate/severe) chronic periodontitis. Another example relates to the oral cancer patients, where some patient samples included patients with a recent diagnosis of cancer prior to treatment, while other samples represented cancer survivors who had been treated. This begs the question whether or not it makes sense, and whether it is justified, to put different subtypes of patients within one overall category. However, if such heterogeneity exists between groups of patients in the same category, it is still possible to look at the studies separately, since the estimates of the PI dimension are reported for each included individual study as displayed in Figure 3 and Table 1.

The 95% CIs of the mean scores for the three dental disorders were not presented. Therefore, even though it was visualised that the patients with dental anxiety had the highest pooled mean score and the patients with periodontitis had the lowest pooled mean score, it was impossible to report whether the difference was statistically significant. This is because the 95% CI values of individual patient samples on a 0 to 8 metric converted from the OHIP‐49 may have less range than the 95% CI values of the same patient samples from the OHIP‐14 due to the method we used. Therefore, we think the 95% Cl values of individual patient samples from the OHIP‐14 are too heterogeneous to be meaningfully combined with the converted 95% CI values from the OHIP‐49. This is another limitation of the study.

Further, only patients with one of the three types of dental disorders (dental anxiety, periodontitis and oral cancer) were included in the present study. Patients with other dental disorders, for example, temporomandibular disorders (TMDs) or edentulous patients, may be more likely to have psychosocial disorders than those with periodontitis. However, those patients were not included in the current study because it was pre‐assumed that they would be affected more by items from one of the other dimensions. Therefore, studies on TMD patients were assigned to the review on the Orofacial Pain dimension and studies on edentulous patients were assigned to the review on the Oral Function dimension. This is another limitation of the present review. Future studies are recommended to focus on the PI of the patients with broader dental disorders, such as TMDs and edentulism.

## 5 CONCLUSION

In summary, within its limitations, the present study provides standardised information about the PI dimension, within the proposed four‐dimensional model of OHRQoL, for three dental patient groups (dental anxiety, periodontitis and oral cancer). Results indicate that dental anxiety may have the strongest effect on the PI dimension, while periodontitis may have the weakest effect on the PI dimension among the three dental disorders. Future studies need to confirm whether the reported differences on the PI dimension between the three dental disorders are statistically significant and clinically relevant.

## CONFLICT OF INTEREST

The authors report no conflict of interest.

## AUTHOR CONTRIBUTIONS

NS contributed to concept, study design, full‐text assessment, risk of bias assessment and interpretation of results. AvW and CM contributed to concept, study design, full‐text assessment and risk of bias assessment. All authors critically revised the manuscript and provided final approval before submission.

### Peer Review

The peer review history for this article is available at https://publons.com/publon/10.1111/joor.13064.
